# Effects of optimism on motivation in rats

**DOI:** 10.3389/fnbeh.2015.00032

**Published:** 2015-02-25

**Authors:** Rafal Rygula, Joanna Golebiowska, Jakub Kregiel, Jakub Kubik, Piotr Popik

**Affiliations:** ^1^Affective Cognitive Neuroscience Lab, Department of Behavioral Neuroscience and Drug Development, Institute of Pharmacology Polish Academy of SciencesKrakow, Poland; ^2^Faculty of Health Sciences, Collegium Medicum, Jagiellonian UniversityKrakow, Poland

**Keywords:** rat, motivation, ambiguous-cue interpretation, progressive ratio, pessimism, optimism, cognitive judgment bias

## Abstract

In humans, optimism is a cognitive construct related to motivation; optimists exert effort, whereas pessimists disengage from effort. In this study, using a recently developed ambiguous-cue interpretation (ACI) paradigm we took the unique opportunity to investigate whether “optimism” as a trait is correlated with motivation in rodents. In a series of ACI tests (cognitive bias screening, CBS), we identified rats displaying “pessimistic” and “optimistic” traits. Subsequently, we investigated the trait differences in the motivation of these rats to gain reward and to avoid punishment using a progressive ratio (PR) schedule of reinforcement paradigm. Although “optimistic” and “pessimistic” animals did not differ in their motivation to avoid punishment, the “optimistic” rats were significantly more motivated to gain reward than their “pessimistic” conspecifics. For the first time, we showed an association between cognitive judgment bias and motivation in an animal model. Because both investigated processes are closely related to mental health and wellbeing, our results may be valuable for preclinical modeling of many psychiatric disorders.

## Introduction

Optimism is a cognitive construct consisting of expectancy, and it has motivational implications (Carver and Scheier, 2014). If an individual is confident about eventual success (optimistic), effort continues. When the individual is doubtful (pessimistic), there is a tendency to disengage effort (Carver and Scheier, 2014). Given the origin of optimism in a broad view of motivation, it is natural that researchers have investigated its role in motivation-relevant outcomes. In humans, optimism has been linked to a greater likelihood of completing college (Solberg Nes et al., 2009), better balancing of effort expended (Segerstrom and Nes, 2006), an increase in effort when circumstances are favorable and a decrease in effort when circumstances are unfavorable (Pavlova and Silbereisen, 2013), and a tendency to increase goal engagement for high-priority goals (Geers et al., 2009). Optimistic individuals have also been shown to display greater engagement in treatment programs (nutrition, education and psychotherapy) and, consistent with their greater engagement in other high-priority tasks (Geers et al., 2009; Carver and Scheier, 2014), to work harder at their relationships (Segerstrom, 2007; Rand, 2009).

Given that optimism is beneficial in many life domains, it is surprising how little attention it has received in behavioral neuroscience. Although several reports over the past decade have indicated that the cognitive judgment biases of “optimism” and “pessimism” can be measured in animals following various behavioral and pharmacological manipulations (Harding et al., 2004; Brilot et al., 2010; Enkel et al., 2010; Mendl et al., 2010; Bateson et al., 2011; Doyle et al., 2011; Bethell et al., 2012; Rygula et al., 2012), almost none of them investigated cognitive judgment bias as an enduring and stable behavioral trait that could be used to evaluate its motivational implications.

We have shown recently that in animals, similar to in humans, cognitive judgment bias has components of both enduring traits and transient states (Rygula et al., 2013). A trait captures a stable individual level of pessimism/optimism that is generally experienced, whereas a state represents the valence of cognitive judgment bias that may change based on the situation or contextual factors (Rygula et al., 2013). Following this assumption, in the present study, we isolated 2 groups of rats that consistently differed in their cognitive judgment bias over time. These 2 groups of “pessimists” and “optimists” were subsequently tested for their motivation to gain reward (approach motivation) and to avoid punishment (avoidance motivation) in a paradigm using a progressive ratio (PR) schedule of reinforcement (Hodos, 1961; Hodos and Kalman, 1963). In this paradigm, the number of responses required to deliver the reinforcer increases progressively, and the traditional measure of the subject’s motivational state is the ratio at which responding ceases within a time-limited experimental session, the breakpoint (Ferguson and Paule, 1997; Bowman and Brown, 1998; Barr and Phillips, 1999).

In our study, the rats were required not only to make an increasing number of responses to generate reward delivery (motivation to gain reward) but also to avoid electric foot shock (motivation to avoid punishment).

We hypothesized that in rats, similar to humans, the traits of “optimism” and “pessimism” would be associated and correlated with motivation to gain reward and avoid punishment.

## Methods

### Ethics statement

All described experimental procedures were conducted in accordance with the NIH Guide for the Care and Use of Laboratory Animals and were approved by the Committee for Ethics in Animal Experiments at the Institute of Pharmacology Polish Academy of Sciences.

### Subjects and housing

We used 80 male *Sprague Dawley* rats (Charles River, Germany) weighing between 175–200 g upon arrival. The animals were housed in groups of 4, in a temperature (21 ± 1°C) and humidity (40–50%) controlled room under a 12/12 h dark/light cycle (lights on at 06:00 h). The animals were habituated to the housing conditions and experimental facility for 2 weeks after arrival and before the start of experiments. In all of the experiments, the animals received 15–20 g of food per rat per day (standard laboratory chow) what corresponds to mild food-deprivation. The food deprivation began 7 days prior to beginning of the training. The water was provided *ad libitum*. The animals were trained and tested during the light phase of the dark/light cycle. The rats were habituated to the experimental room for 30 min prior to training and testing sessions and were tested once daily.

### Apparatus

The experiments were conducted in 8 computer-controlled, operant conditioning boxes (Med Associates, St Albans, Vermont, USA); the boxes were equipped with lights, speakers, liquid dispensers (0.1 ml of 5% sucrose solution), electric grid floors, and 2 retractable levers. The levers were located on both sides of the liquid dispenser. The experimental protocols were written in Med State notation code (Med Associates).

### Behavioral training

The experimental training and testing procedures for the ambiguous-cue interpretation (ACI) paradigm used in this study were modified from procedures previously described by Enkel et al. (2010) and have been described in detail elsewhere (Rygula et al., 2012, 2013, 2014a,b,c, 2015; Papciak et al., 2013).

In brief, initially the animals were trained to press one lever when a “positive” tone (2000 Hz at 75 dB) signaled reward (5% sucrose solution) availability, and to press second lever when another, “negative” tone (9000 Hz at 75 dB) signaled a forthcoming punishment (0.5 mA, 10 s). By pressing appropriate levers the animals could either receive a reward or avoid punishment. The tone presentations were separated by 10 s intertrial intervals (ITI) and each training session lasted 30 min. The animals had to fulfill the criteria of at least 90% of accurate responses to the tone signaling reward availability maintained over 3 consecutive training sessions and, at least 60% of correct punishment-prevention responses maintained over 3 consecutive training sessions, to proceed to the discrimination training.

During the discrimination-training phase, the animals were trained to discriminate between pseudo-randomly presented positive (20) and negative (20) tones, by responding to the appropriate levers (as learned in the previous training stages) to minimize punishment and maximize reward delivery. Each discrimination training session lasted 40 min. The animals had to achieve a minimum of 70% correct responses with each lever, maintained over 3 consecutive discrimination sessions to be qualified for the ACI testing.

### Ambiguous-cue testing

During the ACI testing sessions the animals were exposed to 20 negative, 20 positive, and 10 ambiguous (5000 Hz at 75 dB) tone presentations. The tones were played in a pseudo-randomized order and were separated by 10 s ITIs. The responses to each tone (positive, ambiguous and negative) during the ACI testing were analyzed as the proportion of the overall number of responses to a given tone. To calculate the cognitive bias index we subtracted the proportion of negative responses to the ambiguous-cues from the proportion of positive responses to the ambiguous-cue, what resulted in values ranging between −1 and 1. The values above 0 indicated an overall positive judgment and “optimistic” interpretation of the ambiguous-cue while the values below 0 indicated overall negative judgment and “pessimism”.

### Cognitive bias screening (CBS)

The cognitive bias screening (CBS) procedure has been described in detail elsewhere (Rygula et al., 2013). In brief, to assess the cognitive judgment bias as a trait, we examined the animals in a series of 10 consecutive ACI tests conducted at 1-week intervals. Based on the average (AVG) cognitive bias index obtained from these 10 ACI tests, the rats were divided into 2 subgroups: “optimistic” and “pessimistic”.

### Measurement of motivation under the progressive-ratio schedule of reinforcement

To assess whether the traits “optimism” and “pessimism” interact in animals with the motivation to gain reward and/or to avoid punishment, the rats were tested for the breakpoints of response on the PR schedules of reinforcement (traditional measure of motivation in laboratory animals).

Because all animals had previously learned that pressing one lever in the operant chamber results in a reward delivery and pressing the other lever prevents punishment, they were switched directly after the CBS procedure to the tests with a progressive-ratio schedule of reinforcement.

#### Measurement of the motivation to gain reward—progressive ratio schedule of reinforcement

At the beginning of the PR session, both levers were extended into the operant chamber, and each rat received one non-contingent, experimenter-delivered reward (0.1 ml of 20% sucrose solution). Later during the session, responding on the previously rewarded “positive” lever was reinforced with sucrose solution delivery, and the number of responses required to produce the next reward increased progressively with each successive reward obtained. The steps of the exponential progression used in our study were the same as those previously developed by Roberts and Bennett (1993) and previously used by Solinas et al. (2003) for food reinforcement and were based on the following equation: response ratio = (5eX^(0.2 × reward number)^)−5, rounded to the nearest integer. Thus, the values of the steps were 1, 2, 4, 6, 9, 12, 15, 20, 25, 32, 40, 50, 62, 77, 95, 118, 145, 178, 219, 268, 328, 402, 492, 603, etc. Each reward delivery was accompanied by presentation of the tone (5 s, 2000 Hz at 75 dB sound pressure level (SPL)) previously used in the ACI paradigm to signal reward availability. After each reward delivery, both levers retracted for a 10 s ITI. Sessions lasted 30 min. Responding on the “negative” lever, previously associated with avoiding of the punishment, was recorded but had no consequences.

#### Measurement of the motivation to avoid punishment—progressive ratio schedule of reinforcement

One week after measurements of motivation to gain reward, the animals were subjected to tests of motivation to avoid punishment in a modified PR paradigm. In this paradigm, extension of the levers was accompanied by presentation of the tone (9000 Hz at 75 dB SPL), which previously, in the ACI paradigm, signaled a forthcoming punishment. The rats had to make a progressively increasing number of responses on the “negative lever”, which was previously (in the ACI paradigm) associated with avoiding punishment, to avoid an electric shock (0.5 mA, 10 s). The steps of the exponential progression were the same as those used previously for measuring motivation to gain reward. Each consecutive lever press during the tone presentation prolonged its duration by 10 s, until the animal reached the number of lever presses required to avoid punishment at that stage. Reaching the required number of lever presses resulted in tone termination, retraction of the levers and a 10 s ITI. Not reaching the required number of lever presses resulted in prolongation of the tone and shock delivery. Pressing the lever after the shock onset terminated the tone and shock and initiated a 10 s ITI. After each shock delivery, both levers retracted for a 10 s ITI. Sessions lasted 30 min. Responding on the other “positive” lever was recorded but had no consequences.

### Experimental design and behavioral measures

**Figure 1:**
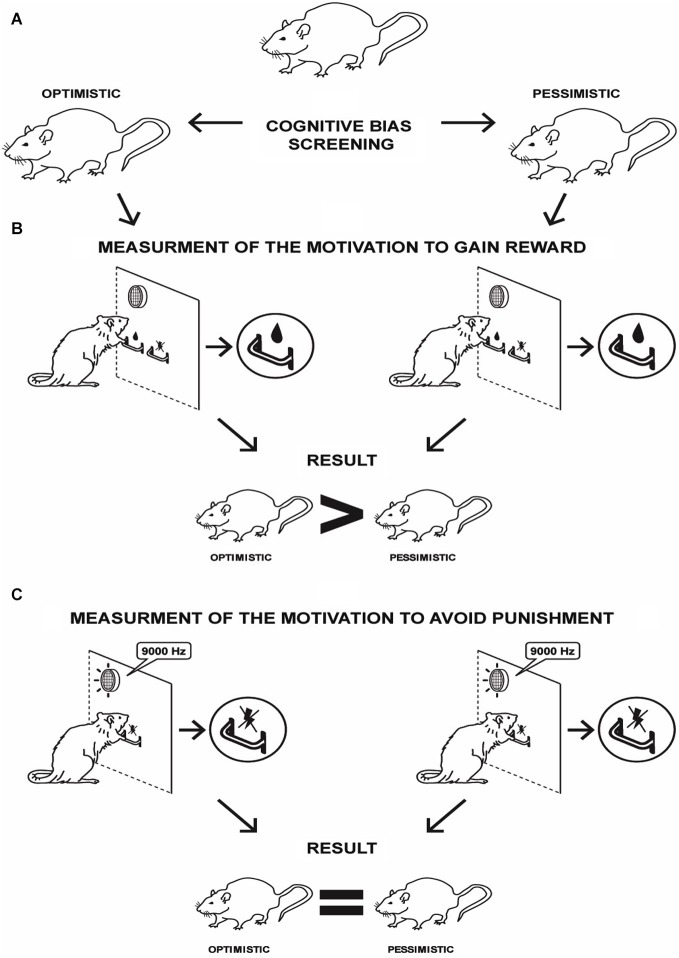
**Schematic representation of the experimental schedule and main result of the study. (A)** Cognitive Bias Screening enabled the separation of 2 groups of animals that were clearly distinct in their interpretations of the ambiguous cues over time: “optimistic” (*N* = 24, AVG cognitive bias index > 0) and “pessimistic” (*N* = 56, AVG cognitive bias index < 0). **(B)** Measurement of motivation to gain reward. On the progressive ratio (PR) schedule of reinforcement, “optimism” has been associated with a significantly higher motivation to gain reward than “pessimism”. **(C)** Measurement of motivation to avoid punishment. No significant differences were observed in the motivation to avoid punishment between the “optimistic” and “pessimistic” animals.

The experimental design is schematically presented in Figure 1. After attaining a stable discrimination performance (more than 70% correct responses to each tone over 3 consecutive days), each rat was subjected to the CBS procedure as previously described (Rygula et al., 2013). After establishing the “optimistic” and “pessimistic” traits in the individual animals, the rats were divided into 2 experimental groups: “optimistic” and “pessimistic” (Figure 1A). To assess whether the traits of “optimism” and “pessimism” interact with motivation, the rats were tested for the breakpoints of response on the PR schedule of reinforcement. In the first test (Figures 1B, 4A–D), performed 24 h after the final ACI test, the animals were required to make an increasing number of the positive lever presses to gain sweet sucrose rewards (identical to that used in the ACI paradigm). In the second test (Figures 1C, 5A–E), performed 7 days later, the animals were required to make an increasing number of the negative lever presses to avoid mild electric foot-shock (punishment used previously in the ACI paradigm).

**Figure 2:**
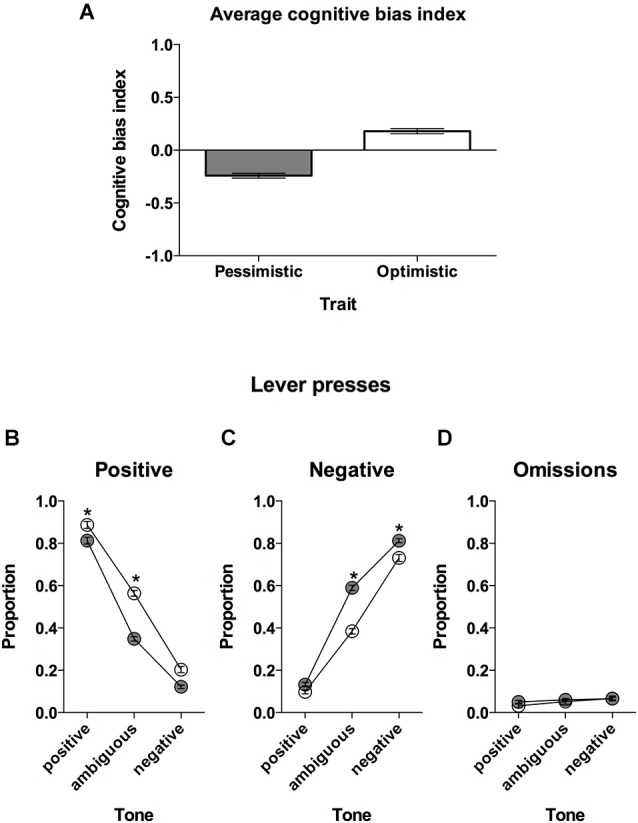
**“Optimistic” vs. “pessimistic” animals; results of the cognitive bias screening. (A)** The mean ± SEM cognitive bias index of the animals classified (based on 10 ACI tests) as “optimistic” (open bar, *N* = 24) vs. “pessimistic” (filled bar, *N* = 56). A cognitive bias index above 0 indicates an overall positive judgment and “optimistic” interpretation of the ambiguous cue. **(B)** The mean ± SEM proportions of positive, **(C)** negative and **(D)** omitted responses to the trained and ambiguous tones in the “optimistic” (open circles, *N* = 24) and “pessimistic” (filled circles, *N* = 56) rat groups. * indicates significant (*p* < 0.05) differences between the “optimistic” and “pessimistic” animals.

**Figure 3:**
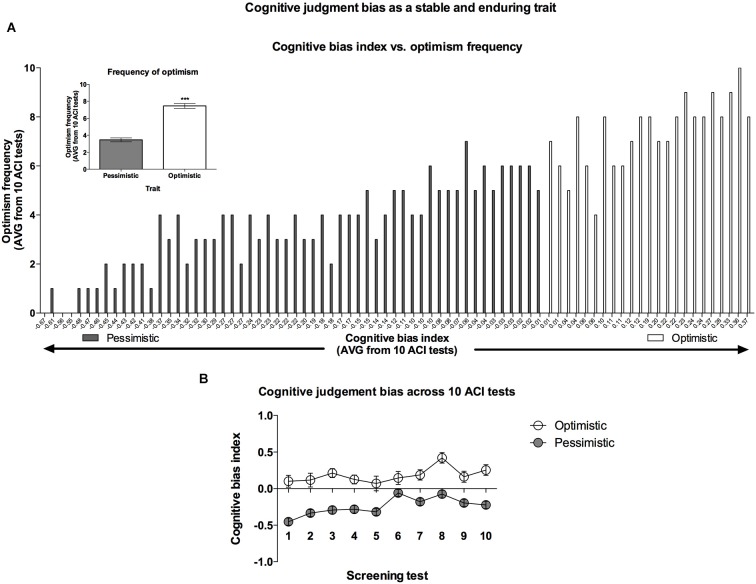
**Cognitive bias as a stable and enduring behavioral trait. (A)** Histogram of the “optimism” frequency (number of ACI tests resulting in a cognitive bias index above 0 out of the 10 ACI tests comprising the cognitive bias screening) in relation to the valence of individual cognitive bias index (AVG from cognitive bias screening) in all (*N* = 80) animals. In the inset: The mean ± SEM “optimism” frequency of the animals classified (based on 10 ACI tests) as “optimistic” (open bar, *N* = 24) and “pessimistic” (filled bar, *N* = 56). **(B)** The mean ± SEM cognitive bias index of the animals classified as “optimistic” (open circles, *N* = 24) and “pessimistic” (filled circles, *N* = 56) across all 10 baseline ACI tests.

**Figure 4:**
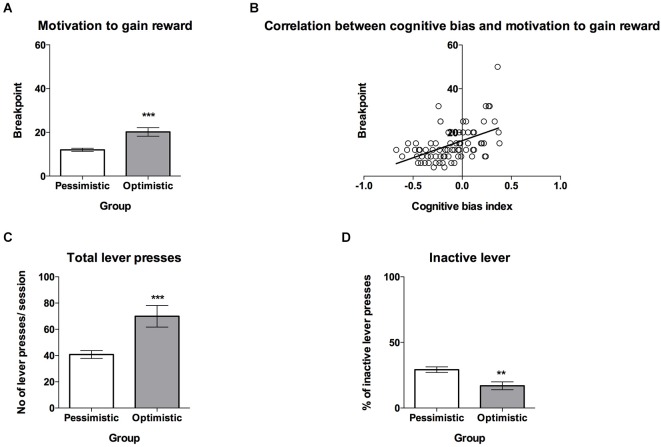
**The “optimistic” rats are significantly more motivated to gain reward than their “pessimistic” conspecifics. (A)** The mean ± SEM breakpoint reached on the PR schedule of reinforcement (sweet sucrose solution as a reward) by animals classified (based on 10 ACI tests) as “optimistic” (open bar, *N* = 24) vs. “pessimistic” (filled bar, *N* = 56). **(B)** The correlation between cognitive bias index and motivation to gain reward, measured by the PR schedule of reinforcement (*N* = 80) **(C)** The mean ± SEM for the number of total lever presses made during one PR schedule of reinforcement test session by animals classified as “optimistic” vs. “pessimistic”. **(D)** The mean ± SEM percentage of inactive lever presses made during one PR schedule of reinforcement test session by animals classified as “optimistic” vs. “pessimistic”. *** indicates significant (*p* < 0.001) differences between the “optimistic” and “pessimistic” animals.

**Figure 5:**
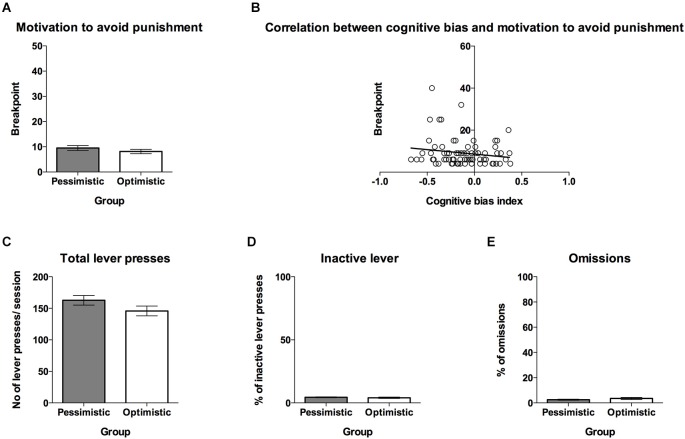
**The “optimistic” and “pessimistic” rats do not differ in their motivation to avoid punishment. (A)** The mean ± SEM breakpoint reached on the PR schedule of reinforcement (electric foot shock as an avoidable punishment) for animals classified (based on 10 ACI tests) as “optimistic” (open bar, *N* = 24) vs. “pessimistic” (filled bar, *N* = 56). **(B)** The correlation between cognitive bias index and motivation to avoid punishment, measured by the PR schedule of reinforcement (*N* = 80) **(C)** The mean ± SEM number of total lever presses made during one PR schedule of reinforcement test session by animals classified as “optimistic” vs. “pessimistic”. **(D)** The mean ± SEM percentage of inactive lever presses made during one PR schedule of reinforcement test session by animals classified as “optimistic” vs. “pessimistic”. **(E)** The mean ± SEM percentage of omitted responses during one PR schedule of reinforcement test session by animals classified as “optimistic” vs. “pessimistic”. * indicates significant (*p* < 0.05) differences between the “optimistic” and “pessimistic” animals.

### Statistics

We analyzed the data using SPSS (version 20.0, SPSS Inc., Chicago, IL, United States). The distribution of the cognitive bias index data was tested using the Kolmogorov-Smirnov test. The difference between “optimists” and “pessimists” in the frequency of “optimism” was analyzed using the t-test. The differences in the processing of the experimental tones between the “optimists” and “pessimists” were investigated using 4-way analysis of variance (ANOVA) with the between-subject factor of Cognitive judgment bias (2 levels: optimistic and pessimistic) and the within-subjects factors of Test (10 levels: baseline test 1–10), Lever (2 levels: positive and negative) and Tone (3 levels: positive, ambiguous and negative). The differences between “optimistic” and “pessimistic” animals in motivation to gain reward and to avoid punishment were analyzed separately using *t*-tests. Finally, Pearson correlations between cognitive bias index and motivation to gain reward and avoid punishment were determined. For pair-wise comparisons, we adjusted the values using Sidak’s correction factor for multiple comparisons (Howell, 1997). All of the tests of significance were performed at *α* = 0.05. We tested the homogeneity of variance using Levene’s test and for repeated-measures analyses, we confirmed the sphericity using Mauchly’s test. The data are presented as the mean ± SEM.

## Results

All trained animals (80) reached the training criteria, and qualified for CBS. The “optimists” reached the criteria of positive tone, negative tone and discrimination trainings after 5.7 ± 0.33, 6.25 ± 0.71 and 35.88 ± 1.9 days, respectively, whereas the “pessimists” reached the criteria after 6.23 ± 0.25, 7.16 ± 0.60 and 32.50 ± 0.80 days, respectively. We observed no significant differences in the total duration of the training between the “optimists” and “pessimists” (*t*_(78)_ = −0.85, NS).

The AVG cognitive bias index of all of the experimental animals established based on the CBS was −0.11 ± 0.028. The distribution of the cognitive bias index data during CBS (Figure 3A) was normal (*Z* = 0.98, *N* = 80, Kolmogorov-Smirnov test).

An analysis of the response of animals to the positive and negative levers following reference and ambiguous tones across the screening period indicated no test-retest effects. Although the Test × Lever × Tone interaction was significant (*F*_(18,1404)_ = 4.50, *p* < 0.001), *post hoc* pairwise comparisons revealed that between-test differences showed no unequivocal pattern.

We observed no regularity in the distribution of the AVG cognitive bias index within the cages. In seven cages, all of the animals were “pessimists”; in two cages, 3 animals were “optimists” and 1 was “pessimist”; in seven cages, 2 rats were “optimists” and 2 were “pessimists”; and in four cages, 1 rat was “optimistic” and 3 rats were “pessimistic”.

### “Pessimistic” vs. “optimistic” rats

Based on the results of the CBS (Figure 2A) we divided the animals into 2 groups that were clearly distinctive in the interpretation of the ambiguous-cues over time: “optimists” (*N* = 24, AVG cognitive bias index > 0) and “pessimists” (*N* = 56, AVG cognitive bias index < 0).

Further analysis revealed significant differences in the patterns of response between “optimists” and “pessimists” (Lever × Tone × Cognitive bias interaction (*F*_(2,156)_ = 24.83, *p* < 0.001)). The “optimists” responded significantly less often to the negative lever in response to the ambiguous tone compared to “pessimists” (*p* < 0.001, Figure 2C). In contrast, in response to the ambiguous tone, the animals classified as “pessimistic” responded significantly less often to the positive lever (*p* < 0.001, Figure 2B). The “optimists” also responded more often to the positive lever in response to the positive and negative tones (*p* < 0.01 and *p* < 0.001, respectively, Figure 2B) and less often to the negative lever in response to the negative tone (*p* < 0.001, Figure 2C).

“Optimists” and “pessimists” did not differ in the numbers of omitted trials (no significant effect of Cognitive bias or Cognitive bias × Tone interaction); however, all of the rats made significantly more omissions following negative and ambiguous tones (*p* < 0.001 and *p* < 0.05, respectively) compared to after the positive tones (significant effect of Tone (*F*_(2,156)_ = 12.85, *p* < 0.001, Figure 2D).

The “optimists” showed an AVG cognitive bias index ranging from 0.01 to 0.38, whereas the cognitive bias index in the “pessimists” ranged from −0.01 to −0.67 (Figure 3A).

Analysis of the “optimism” frequency (number of tests when the cognitive bias index of an individual animal was higher than zero, out of the 10 CBS sessions) revealed that on AVG, rats classified as “optimists” were significantly more frequently “optimistic” than their “pessimistic” conspecifics (Figure 3A—inset).

Although, the cognitive bias index of all rats varied from test to test (significant Test × Lever × Tone interaction (*F*_(18,1404)_ = 4.50, *p* < 0.001)), the differences between the “optimists” and “pessimists” did not change significantly across the CBS (no significant Test × Cognitive bias interaction; *F*_(9,702)_ = 0.73, NS), indicating stability of the traits (Figure 3B).

### Motivation to gain reward and to avoid punishment

On a PR schedule, “optimism” has been found to be associated with a significantly higher motivation to gain reward than “pessimism”. *T*-test analysis revealed that the breakpoints of the “optimistic” animals were significantly higher than those of their “pessimistic” counterparts (Figure 4A, *t*_(78)_ = 4.58, *p* = 0.002). Correlation analysis revealed that the cognitive bias index and motivation to gain reward, measured for the PR schedule of reinforcement, were significantly positively correlated (Figure 4B, *r* = 0.49, *N* = 80, *p* < 0.001). The “optimistic” and “pessimistic” animals also differed significantly in the numbers of total lever presses (Figure 4C, *t*_(78)_ = 4.13, *p* < 0.001) and percentages of inactive lever presses made during the PR test session (Figure 4D, *t*_(78)_ = 3.21, *p* < 0.01).

No significant differences were observed in the motivation to avoid punishment between the “optimistic” and “pessimistic” animals (Figure 5A, *t*_(78)_ = 0.61, NS). There was also no significant correlation between cognitive bias index and motivation to avoid punishment (Figure 5B, *r* = −0.16, *N* = 80, NS). The “optimists” and “pessimists” did not significantly differ in the number of total lever presses (Figure 5C, *t*_(78)_ = 1.33, NS), percentage of inactive lever presses (Figure 5D, *t*_(78)_ = 0.61, NS) and omissions made during the PR test session (Figure 5E, *t*_(78)_ = 1.20, NS).

On AVG, all animals were found to be significantly (*t*_(79)_ = 7.77, *p* < 0.001) more motivated to gain a reward (AVG breakpoint 14.44 ± 0.88) than to avoid punishment (AVG breakpoint 9.10 ± 0.74).

## Discussion

In the present study, we used an animal model to examine whether the traits of “optimism” and “pessimism” are associated with different levels of motivation in rats. Our results indicate that animals displaying the “optimistic” trait were more motivated to obtain a sweet sucrose reward compared to their “pessimistic” conspecifics. There was also a significant positive correlation between the level of “optimism” and motivation to obtain the reward, whereas motivation to avoid punishment did not differ between “optimistic” and “pessimistic” animals.

In combination with our previous report (Rygula et al., 2013), the results of the present study demonstrate clearly that in rats, the valence of cognitive judgment bias is an enduring behavioral trait that may determine other aspects of the animals’ behavior. As shown in Figure 3A, the value of this trait is both quantitative (frequency of “optimism”) and qualitative (height of cognitive bias index). Analysis of the lever responses during tests comprising CBS revealed that the animals classified as “optimistic” were both more “optimistic” (made a significantly higher proportion of positive lever presses in response to the ambiguous cue) and less “pessimistic” (made a significantly lower proportion of negative lever presses in response to the ambiguous cue) than their “pessimistic” conspecifics. This pattern was similar to the one described previously (Rygula et al., 2013). Interestingly, a majority of the rats in the tested cohort (56 out of 80) were “pessimistic”. Further studies with larger samples should determine whether, contrary to humans (Sharot et al., 2011), rats as a species are generally “pessimistic”.

Motivation is a pervasive and important determinant of behavior. It has been conceptualized as selecting goals based on their predictive value, initiating behavior to achieve goals, and maintaining goal-directed action (Dickinson and Balleine, 1994; Wigfield and Eccles, 2000). Philosophers and scientists have discussed approach and avoidance motivation for thousands of years. Elliot and Covington (2001) noted that formal discussion of these concepts dates at least to Democritus (460–370 B.C.). According to Elliot (1999), approach and avoidance motivations differ as a function of valence: in approach motivation, behavior is instigated or directed by a positive/desirable event or possibility, whereas in avoidance motivation, behavior is instigated or directed by a negative/undesirable event or possibility. In our study, to evaluate the approach and avoidance motivation, we applied one of the traditional measures of subjects’ motivational state used in behavioral neuroscience—the breakpoint—assessed by a PR schedule of reinforcement (Ferguson and Paule, 1997; Bowman and Brown, 1998; Barr and Phillips, 1999). The rationale for this measure is that the breakpoint is presumed to reflect a situation in which the “anticipated” effort required to obtain the next reinforcer has become sufficiently great that the animal stops responding. Since Hodos et al. first introduced them in 1961 (Hodos, 1961; Hodos and Kalman, 1963), PR schedules have become one of the most frequently employed tests for alterations in the motivational states of animals. To our knowledge, however, they have never before been used to measure avoidance motivation. This is most likely due to the difficulty in training animals to make an operant response in the presence of a threat when they must overcome fear reactions such as freezing. Because the rats used in our study had been successfully trained previously (in the ACI paradigm) to press a lever to avoid punishment when a tone signaled forthcoming punishment, we have used this unique opportunity to measure their avoidance motivation in the PR schedule of reinforcement. Moreover, because the punishment and reward used in the ACI and PR paradigms were identical, we were able to transfer the animals directly from one paradigm to the other without additional training.

The concepts of optimism and pessimism concern an individual’s expectations for the future (Carver et al., 2010). These concepts have ties not only to folk wisdom but also to a class of psychological theories of motivation, expectancy-value theories that create a logical basis for the ways in which optimism and pessimism influence behavior. The expectancy-value theory posits that individuals’ expectancies for success and the subjective value that they have for succeeding are important determinants of their motivation to perform different achievement tasks (Wigfield and Eccles, 2000). Atkinson (1957) originally defined expectancies as individuals’ anticipations that their performance will be followed by either success or failure and defined value as the relative attractiveness of succeeding or failing at a task. Based on these principles, when confronting a challenge, optimists should be confident and persistent, and pessimists should be more doubtful and hesitant (Carver and Scheier, 2014). Indeed, the results of our study confirm these predictions. Animals that were previously classified as “optimistic” were significantly more motivated to gain reward than their pessimistic conspecifics. In other words, at the same objective value of reward, its subjective value was higher in animals that had a higher expectancy of success. Along with the expectancy-value theory, the lack of differences in avoidance motivation between “optimistic” and “pessimistic” animals may be explained by the relatively lower value of succeeding in avoiding punishment, which in turn could mask the effects of anticipation of success (“optimism”/“pessimism”). Indeed, the AVG breakpoint reached by ALL animals on the PR schedules of reinforcement was significantly lower when animals pressed a lever to avoid punishment than in the case of lever pressing to gain a reward. Further studies using different magnitudes of reinforcement should determine whether higher values of punishment increase the effects of trait on avoidance motivation.

There are, of course, also limitations to the present study. First, all measurements of cognitive judgment bias and motivation were performed using operant conditioning tasks that required extensive use of punishment to shape the animals’ behavior. Because it is uncertain how the history of such training influenced motivation, it would be desirable to test the association between cognitive judgment bias and motivation using alternative methods. For example, the use of the spatial version of the ACI task with rewards of different magnitude and with no punishment may prove particularly enlightening. Another limitation is that the nature of our studies prevented us from establishing whether it is “optimism” that increases approach motivation or vice versa. The present research clearly demonstrates that cognitive judgment bias is linked to motivational dispositions, and this is consistent with theoretical models that place optimism as a defining feature of motivation (e.g., Carver and Scheier, 2014). However, is high approach motivation a cause or a consequence of optimistic judgment bias? Future experimental research will help to clarify this interesting and potentially important issue. Moreover, although “optimism” was associated only with higher motivation to gain reward, it is possible that “optimists” also exhibit stronger avoidance motivation, but only in certain domains and punishment magnitudes. This is an issue that certainly merits further investigation. Finally, further studies may determine whether a higher breaking point in pressing a positive lever in optimistic rats in order to abtain a reward could be related not only to animals’ motivation, but also to compulsive behavior.

Taken together, using multiple consecutive ACI tests and a PR schedule of reinforcement based tasks, we demonstrate, for the first time, a link between cognitive judgment bias and motivation in an animal model. Based on the present results, one would predict that cognitive judgment bias would be a powerful shaper of approach motivation in other species, including humans. Evidence of this may be useful for developing therapeutic interventions that target motivational deficits associated with depressive disorders. This would suggest, for example, that reinforcing optimistic judgment bias either pharmacologically or by cognitive bias modification therapy could help to abolish motivational deficits, which are a core symptom of depression in humans.

## Author contributions

RR, JG, JK and JK conceived and designed the experiments, JG, JK, JK and RR performed the experiments, RR analyzed the data, RR and PP contributed materials/analysis tools, RR wrote the paper, RR, JG, JK, JK and PP revised the paper critically for important intellectual content and gave final approval of the version to be published.

## Conflict of interest statement

The authors declare that the research was conducted in the absence of any commercial or financial relationships that could be construed as a potential conflict of interest.
